# Progesterone receptor isoforms, agonists and antagonists differentially reprogram estrogen signaling

**DOI:** 10.18632/oncotarget.21378

**Published:** 2017-09-28

**Authors:** Hari Singhal, Marianne E. Greene, Allison L. Zarnke, Muriel Laine, Rose Al Abosy, Ya-Fang Chang, Anna G. Dembo, Kelly Schoenfelt, Raga Vadhi, Xintao Qiu, Prakash Rao, Bindu Santhamma, Hareesh B. Nair, Klaus J. Nickisch, Henry W. Long, Lev Becker, Myles Brown, Geoffrey L. Greene

**Affiliations:** ^1^ Department of Medical Oncology, Dana-Farber Cancer Institute, Boston, Massachusetts, USA; ^2^ Center for Functional Cancer Epigenetics, Dana-Farber Cancer Institute, Boston, Massachusetts, USA; ^3^ Ben May Department for Cancer Research, University of Chicago, Chicago, Illinois, USA; ^4^ Evestra Inc., San Antonio, Texas, USA

**Keywords:** cancer, hormones, estrogen, progesterone

## Abstract

Major roadblocks to developing effective progesterone receptor (PR)-targeted therapies in breast cancer include the lack of highly-specific PR modulators, a poor understanding of the pro- or anti-tumorigenic networks for PR isoforms and ligands, and an incomplete understanding of the cross talk between PR and estrogen receptor (ER) signaling. Through genomic analyses of xenografts treated with various clinically-relevant ER and PR-targeting drugs, we describe how the activation or inhibition of PR differentially reprograms estrogen signaling, resulting in the segregation of transcriptomes into separate PR agonist and antagonist-mediated groups. These findings address an ongoing controversy regarding the clinical utility of PR agonists and antagonists, alone or in combination with tamoxifen, for breast cancer management. Additionally, the two PR isoforms PRA and PRB, bind distinct but overlapping genomic sites and interact with different sets of co-regulators to differentially modulate estrogen signaling to be either pro- or anti-tumorigenic. Of the two isoforms, PRA inhibited gene expression and ER chromatin binding significantly more than PRB. Differential gene expression was observed in PRA and PRB-rich patient tumors and PRA-rich gene signatures had poorer survival outcomes. In support of antiprogestin responsiveness of PRA-rich tumors, gene signatures associated with PR antagonists, but not PR agonists, predicted better survival outcomes. The better patient survival associated with PR antagonists versus PR agonists treatments was further reflected in the higher in vivo anti-tumor activity of therapies that combine tamoxifen with PR antagonists and modulators. This study suggests that distinguishing common effects observed due to concomitant interaction of another receptor with its ligand (agonist or antagonist), from unique isoform and ligand-specific effects will inform the development of biomarkers for patient selection and translation of PR-targeted therapies to the clinic.

## INTRODUCTION

Progesterone receptor (PR) governs estrogen signaling in ER+/PR+ breast cancers by dictating chromatin binding of estrogen receptor alpha (ER) [1–3] and modulating the bioavailability of molecules needed for tumor growth [4]. Approximately eighty percent of all ER+ breast cancers are also positive for PR and while selective ER modulators (SERMs) are routinely used as adjuvant therapy in women with PR+ breast cancers, relatively limited progress has been made in the development of effective PR-targeting therapies in the clinic. Gaps in the mechanistic understanding of the genomic actions of PR isoforms, ligand type (agonists and antagonists) and their intersection with estrogen signaling contribute to suboptimal utilization of PR as a potential therapeutic target in breast cancer.

PR exists as two isoforms PRA and PRB, which are coded from two different transcription start sites of PR gene in the region 11q22-23 [5]. PRB is full length PR and PRA has a truncated amino-terminal domain, which lacks the first 164 amino acids [5–8]. In normal breast epithelium, PRA and PRB are expressed at similar levels and loss of coordinate expression of PRA and PRB is an early event in breast cancer [9–12]. PR homodimers as well as heterodimers of both the PR isoforms are transcriptionally active and they differentially regulate gene expression and cellular phenotypes [11, 13–15]. High PRA to PRB ratios in tumors predict poor patient survival and resistance to selective ER modulator (SERM) tamoxifen therapy [10]. Furthermore, PR isoforms A and B differentially modulate the estrogen-dependent growth of breast tumor xenografts, underscoring that critical PR isoform-specific crosstalk occurs between ER and PR [16].

The other major roadblock to clinical translation of PR-targeting therapies is the lack of highly-specific PR-targeting drugs in the clinic and the paradoxical pro- or anti-tumorigenic effects of PR ligands (agonists and antagonists) in different contexts [17–19]. For example, both PR agonists and antagonists inhibit estrogen-mediated growth of ER+/PR+ breast cancer models [17, 18]. Perhaps not surprisingly, PR antagonists and high-dose agonists have been used with limited success for the treatments in advanced breast cancer settings [17, 20–24]. Similarly, activation and inhibition of PR by agonist progesterone [2] and antagonist CDB4124 [1] respectively, can potentiate responses to tamoxifen but with differing levels of anti-tumor activity; Combination therapies of tamoxifen with PR antagonist but not PR agonist promote tumor regression [1, 2]. Importantly, while agonist-activated PR inhibits estrogen-induced tumor growth in the short term, it might do so at a long-term cost of expanding drug resistant clones, increasing stem cells and receptor-negative populations [25–29]. Additionally, the Million Women Study and Women's Health Initiative trials suggest that low-dose PR agonists such as medroxyprogesterone acetate used in combination with estrogen are associated with an increased risk of invasive breast cancer compared to estrogen alone [30–32]. One of the major difficulties in comparing the results of these studies is that they have been conducted using a variety of natural and synthetic progestins, which presumably could promote varied PR biology. Thus, there is a need to perform side-by-side comparisons of the actions of PR isoforms in response to activation by functionally diverse agonists and antagonists, to determine how isoform-specific modulation of estrogen signaling can be translated into PR-targeted therapies.

Previous studies have reported that in rat uterine cells and 3T3 mouse fibroblasts transfected with estrogen and progestin-responsive vectors, PR mediated modulation of ER transcriptional activity is dependent on PR isoform (PRA *versus* PRB) and ligand type (PR agonists *versus* antagonists). Of the two isoforms, PRA is a stronger repressor of ER transcriptional activity. Similarly, antagonist-occupied PR (unless expressly noted, PR hereafter refers to PRA and PRB mixtures.) is a more effective repressor of ER transcriptional activity than agonist-occupied PR [33–35]. Independent studies in monkey kidney CV-1 fibroblasts, MCF10A cells and breast tumor xenografts [16] support these observations of PR isoform and ligand dependence of ER/PR crosstalk [36–38]. While these studies are informative, they lack mechanistic details or were restricted to artificial vector constructs transiently transfected into models that would not accurately recapitulate breast cancer biology.

Therefore, a better genomic understanding of PR ligand type and isoform-specific activities and their intersection with estrogen signaling is needed for careful patient selection to optimize PR-targeted therapies for breast cancer treatment. Importantly, these insights into combinatorial behaviors of ER/PR crosstalk could contribute to the better management of breast cancers that co-express two or more steroid-responsive nuclear receptors.

## RESULTS

### PRA and PRB have isoform-specific cistromes, interactomes, transcriptomes and phenotypic outcomes

**Figure 1:**
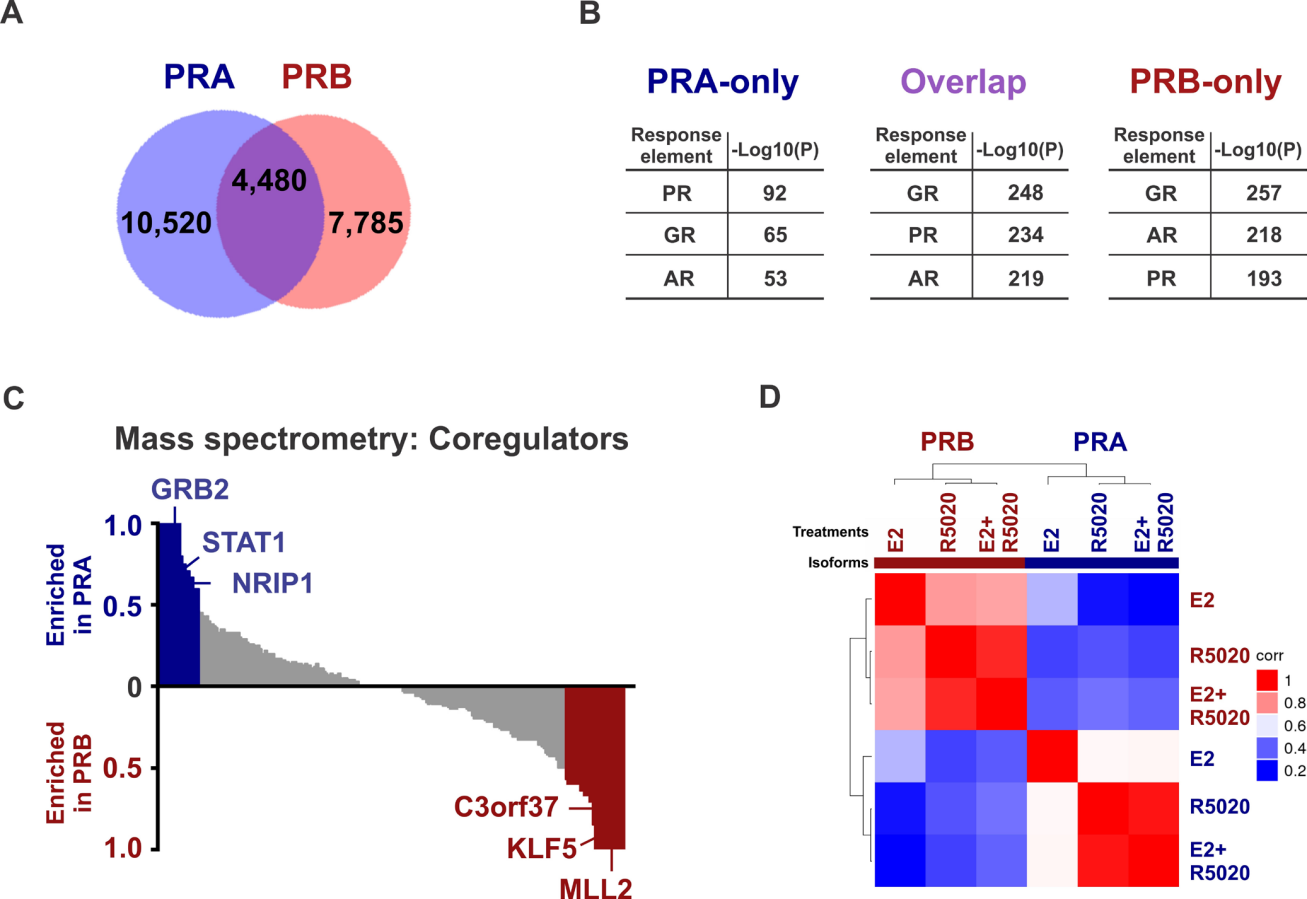
PRA and PRB have isoform-specific cistromes, interactomes, transcriptomes and phenotypic outcomes **A.** Overlap between PRA and PRB binding events in T47D cells treated for 45 minutes with 10 nM R5020. **B.** Top three binding motifs enriched at the binding sites for only PRA, only PRB or overlapping sites for both the receptors. −Log10(P) depicts the significance for the enrichment of a hormone response element in the binding sites of interest. The full list of motifs is provided in Supplementary Table 1. **C.** Mass spectrometry of immunoprecipitates of PR isoform-specific pull downs in T47D cells treated with estrogen plus R5020 identifies distinct interactomes for PRA and PRB. Pull down with control IgG was used as a background control. Two independent experiments were performed. **D.** Unsupervised clustering of sample-sample correlation of transcriptomes observed in T47D cells after 12 hours of treatments with 10 nM E2, R5020 or with both the hormones. High correlation (i.e., correlation coefficient 1) between any two samples is displayed in red and low correlation (i.e., correlation coefficient 0) is displayed in blue.

**Figure 2:**
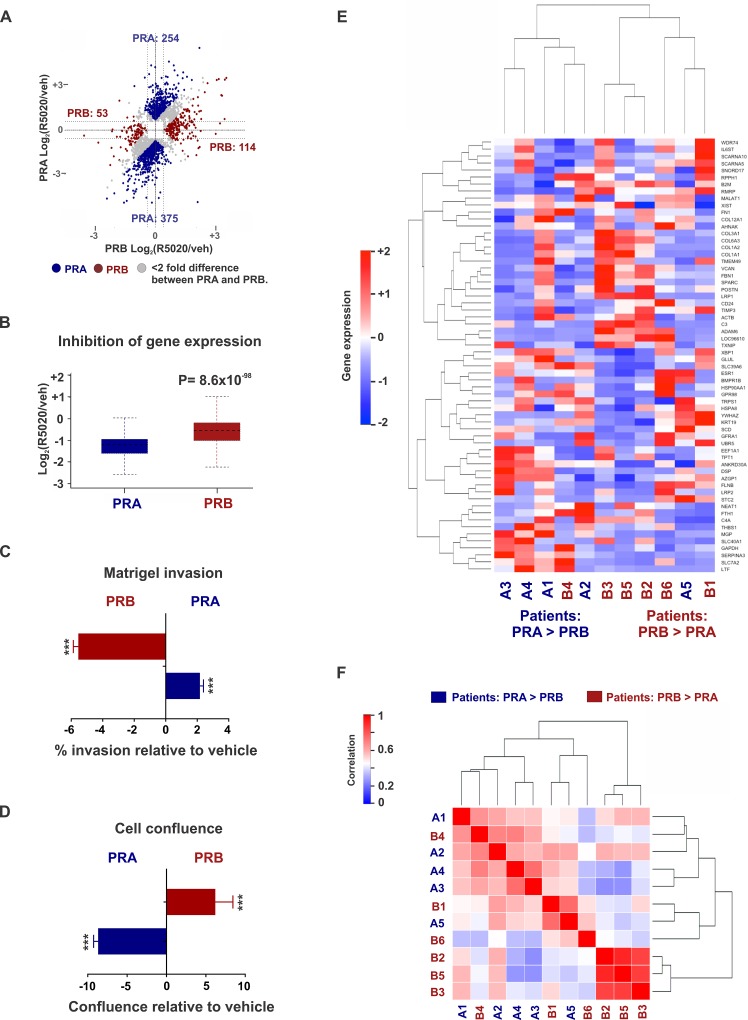
Differential gene expression in patient tumors expressing disproportionate levels of PRA and PRB **A.** Dot plots represent PRA and PRB-regulated genes in T47D cells treated with 10 nM R5020 for twelve hours. PRA is a stronger inducer (or repressor) of gene expression than PRB for the genes represented by blue dots. Conversely, PRB is a stronger inducer (or repressor) of gene expression than PRA for the genes represented by red dots. **B.** Box plots depict ensemble of magnitude of inhibition of gene expression by PRA or PRB. Dot plots and box plots represent union of PRA and PRB-regulated genes. **C.**, **D.** Changes in **C.** matrigel invasion and **D.** cell confluence of ER+/PRA+ and ER+/PRB+ T47D cells in response to treatments with 10 nM R5020. ^***^ denotes *P*-value < 10^-3^. **E.**, **F.** Heatmaps display **E.** gene expression and **F.** sample-sample correlation in five patient tumors expression higher PRA *versus* PRB and six tumors with higher expression of PRB *versus* PRA. High correlation (i.e., correlation coefficient 1) between any two samples is displayed in red and low correlation (i.e., correlation coefficient 0) is displayed in blue. Data for nine out of 11 tumors was obtained from Rojas et al [44]. Data for two other unpublished tumors (tumors B3 and B5) used in this study was kindly provided by Dr. Claudia Lanari and Dr. Martin Abba.

PR isoform-specific ChIP-seq in T47D cells expressing either PRA or PRB on a PR-deficient background suggested that there were 4,480 binding sites common to PRA and PRB, while 10,520 were unique to PRA and another 7,785 sites were specific to PRB (Figure 1A and Supplementary Table 1). Select genomic sites were investigated by directed ChIP-qPCR (Supplementary Figure 1A and 1B). These data highlight the overlapping but distinct nature of isoform-specific cistromes and suggest that PR isoforms are differently recruited to the genome. Both PR isoforms have identical DNA binding domains [5] and as expected, response elements for the nuclear receptor family 3C (NR3C) receptors for progestins, glucocorticoids and androgens were the most highly enriched binding motifs at the PR isoform binding sites (Figure 1B). NR3C receptors share degenerate binding motifs and the enrichment of NR3C motifs suggests a potential for crosstalk within this family of nuclear receptors [39]. The observation that both PR isoforms bound similar motifs (Figure 1B) at mostly different locations (Figure 1A), led us to hypothesize that accessory cofactors might facilitate differential recruitment of PR isoforms. Indeed, Ingenuity analyses of isoform-specific direct targets revealed STAT1 to be enriched at PRA sites alone, while tumor suppressor KLF5 was enriched only for PRB and p53 was found at the binding sites of both isoforms. Mass spectrometry analysis of PRA and PRB-associated cofactors supported these predictions and identified strikingly different sets of co-regulators that are recruited by each of the PR isoforms (Figure 1C, Supplementary Figure 2A - 2B and Supplementary Table 2). For example, cofactors GRB2, STAT1 and NRIP1 preferentially interacted with PRA, whereas MLL2, KLF5 and C3orf37 mainly interacted with PRB, while key epigenomic proteins such as TRIM33, KDM4B and p300 were enriched for both the isoforms (Figure 1C). Importantly, pro-inflammatory pathways were enriched both in PRA-associated transcriptomes (Supplementary Figure 2C) and in PRA interactomes (Supplementary Figure 2D), indicating a preference for PRA to promote inflammation. Based on our findings of distinct isoform-specific cistromes and interactomes and previous reports of differential gene regulation by PR isoforms [14, 40, 41], we hypothesized that selective activation of each PR isoform results in different transcriptional responses. Unsupervised analysis of transcriptional responses in T47D cells expressing PRA or PRB clustered gene expression into PR isoform-specific groups (Figure 1D and Supplementary Table 3). Importantly, out of the two isoforms, PRA was a stronger repressor of gene expression for 375 gene targets while the same was true for only 53 gene targets in the case of PRB (Figure 2A). Also, PRA inhibited gene expression with greater magnitude (Figure 2B), thus collectively indicating that of the two isoforms, PRA is a more potent inhibitor of gene expression. RNA-seq results were validated by RT-PCR of select genes (Supplementary Figures 1C-1E). Our observations highlight the breast cancer relevance of prior studies that reported similar observations using artificial vector constructs transfected in rat uterine cells, 3T3 mouse fibroblasts, monkey kidney CV-1 fibroblasts and MCF10A cells [33–38]. These differences in PR isoform-specific actions were further reflected in Ingenuity analyses of isoform-specific transcriptomes, which predicted that the two PR isoforms regulate cell invasion and proliferation pathways in opposite directions (Supplementary Figure 1F). In agreement with these Ingenuity predictions and prior literature on PR isoforms [6, 7, 16, 42, 43], PRA increased invasion (Figure 2C) and decreased proliferation (Figure 2D), while PRB had the opposite effect on these phenotypes.

Of note, the PR isoform-dependence of transcriptomes and the association between the relative abundance of PR isoform A to breast cancer invasiveness have been observed in independent cell model systems indicating that our observations could be generalizable beyond the T47D model system used in this study [40–43]. To evaluate the clinical relevance of PR isoform-dependent gene expression, we performed transcriptomic analyses in eleven PR+ patient tumors that express high levels of either PR isoform [44]. Of these, five tumors have high PRA:PRB ratios, while the remaining six have low PRA:PRB ratios. Differential gene expression patterns were observed in tumors expressing either higher levels of PRA or PRB (Figure 2E - 2F and Supplementary Table 3) although the clustering signal was weaker compared to that observed in *in-vitro* experiments (Figure 1D), which could be due to tumor heterogeneity, limited sample numbers, and inter-patient variabilities.

In summary, the observed differences in the cistromes, interactomes, transcriptomes and phenotypic outcomes regulated by the two PR isoforms in breast cancer justifies the investigation of isoform-dependent therapeutic targeting of PR in PR+ breast cancers.

### Differential transcriptomes in xenografts treated with various PR agonists and antagonists

**Figure 3:**
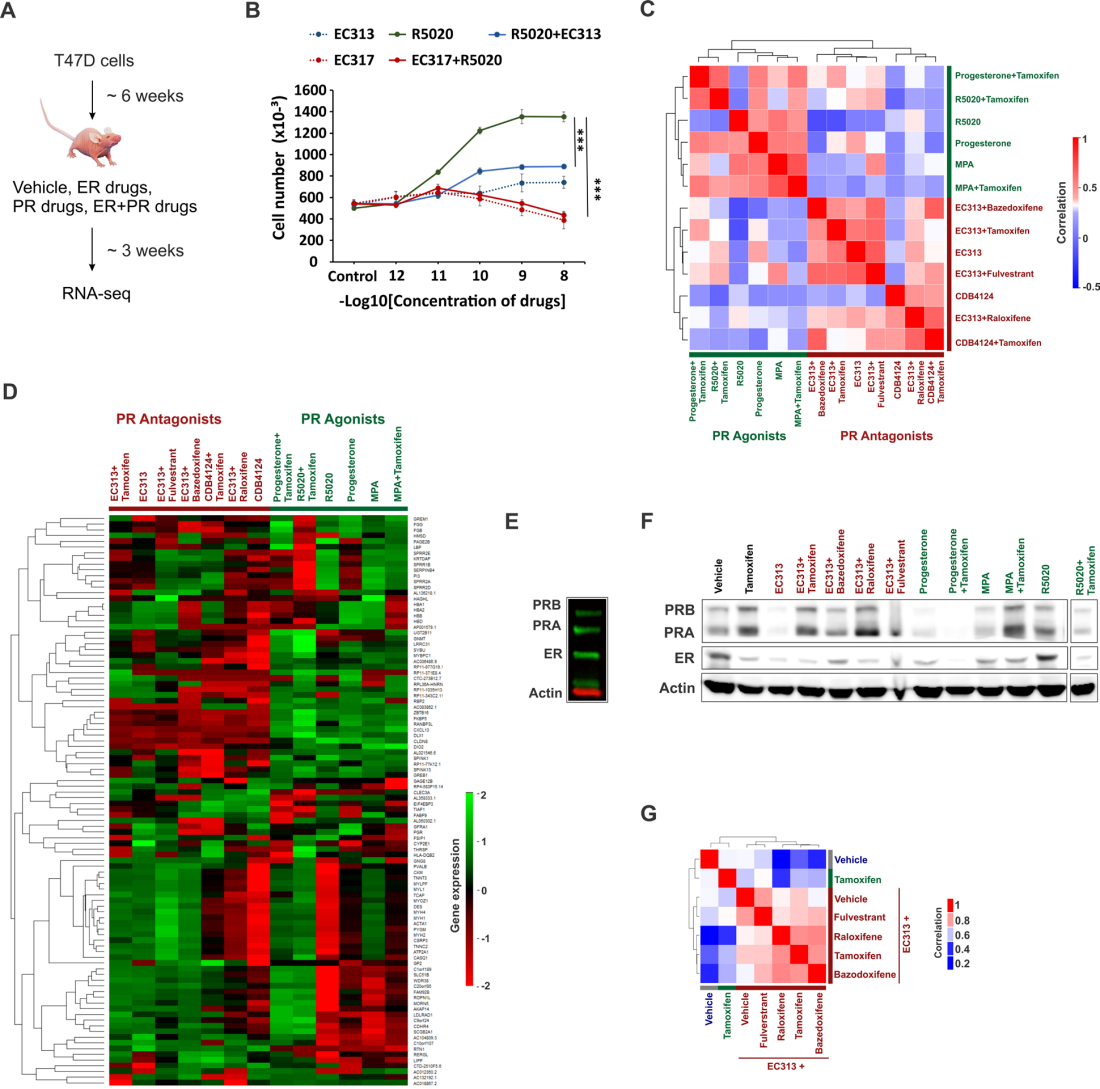
Differential transcriptomes in xenografts treated with various PR agonists and antagonists **A.** T47D xenografts were grown for about 6 weeks and then were subsequently treated with various combinations of vehicle, ER or PR-targeting drugs. Post treatments, xenografts were harvested and RNA-seq was performed. **B.** Cell viability of T47D cells in response to treatments with various combinations of PR agonist R5020, pure PR antagonist EC317 and selective PR modulator (SPRM) EC313. These drugs were treated at various concentrations (1 pM to 10 nM) mentioned on the horizontal axis. Vertical axis represents the cell numbers after the end of six days of treatments of interest. **C.**, **D.** Heatmaps display unsupervised clustering of **C.** sample-sample correlations and **D.** gene expression observed in T47D xenografts treated with various combinations of ER and PR-targeting drugs. **E.** Immunoblots to measure ER and PR levels in T47D cells used to seed T47D xenografts. **F.** Immunoblots to measure ER and PR levels in various T47D xenografts used in the study. The immunoblots for individual and combination (with tamoxifen) therapies with CDB4124 and CDB4453 could not be included because of the lack of the starting material. **G.** Unsupervised clustering of sample-sample correlations observed between transcriptomes of T47D xenografts treated with vehicle, tamoxifen, SPRM EC313 alone or in combination with SERMs tamoxifen, bazedoxifene, raloxifene or selective ER-degrader fulvestrant. High correlation (i.e., correlation coefficient 1) between any two samples is displayed in red and low correlation (i.e., correlation coefficient 0) is displayed in blue.

**Figure 5:**
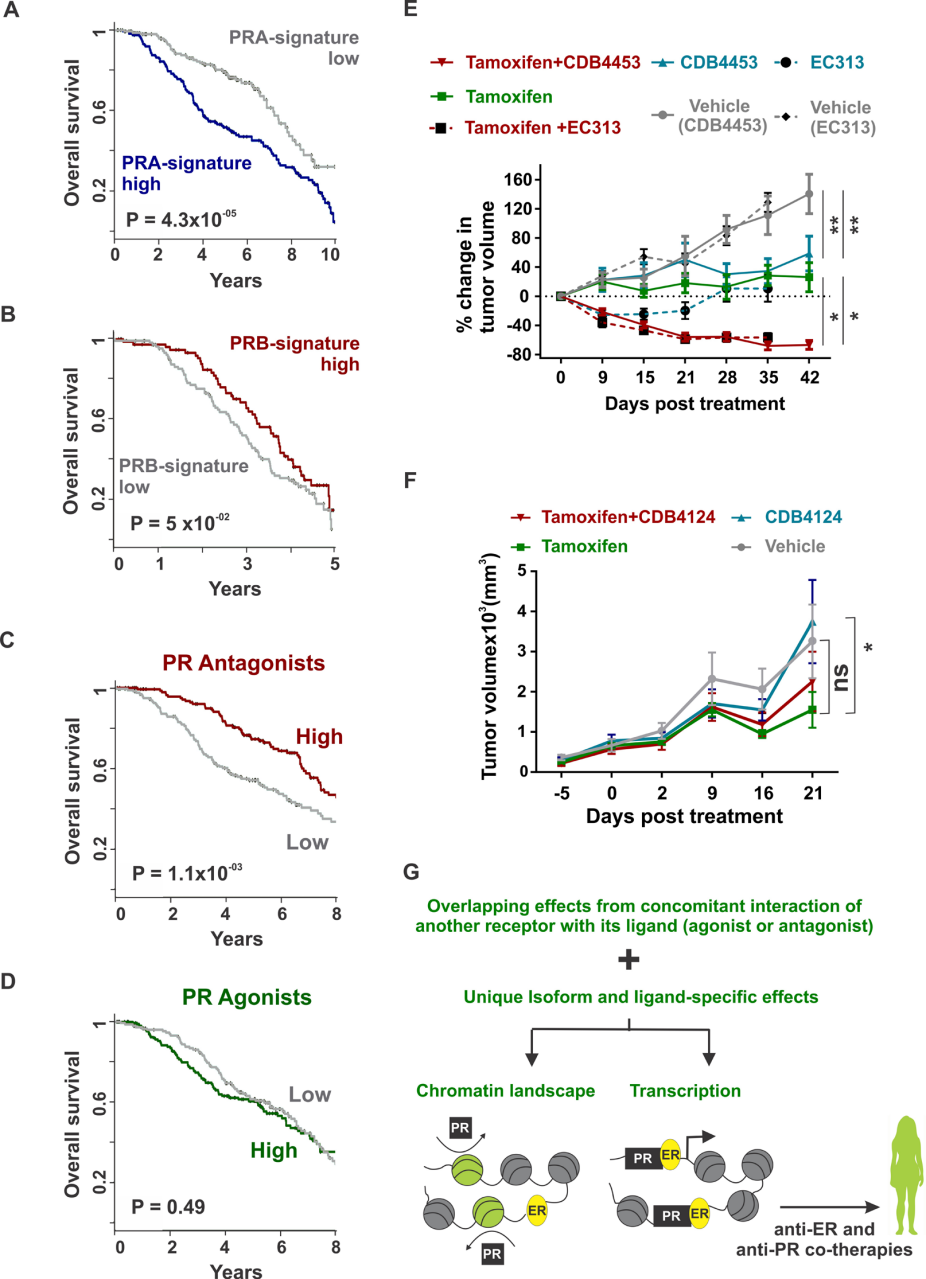
Combination therapies with tamoxifen and SPRMs result in tumor regression (A - B) Overall survival in METABRIC's discovery cohort as classified by high or low expression of **A.** PRA or **B.** PRB-regulated genes. Top 1000 differential PRA and PRB-regulated genes were obtained from Figure 1D and are provided in Supplementary Table 3 **C.** - **D.** Overall survival in METABRIC's discovery cohort as classified by high or low expression of top differential. **C.** PR antagonists or **D.** PR agonists-regulated genes. Top 100 PR agonist and antagonist-regulated genes were obtained from Figure 3C and are provided in Supplementary Table 4. **E.** T47D xenografts were grown in ovariectomized nude mice containing estrogen silastic implants and were treated with placebo, tamoxifen, CDB4453, EC313 or tamoxifen plus CDB4453 or EC313. Average tumor volume at the start of therapies was 125 mm^3^ and percentage change in tumor volume is shown (*n* > 16). **F.** ER+/PR- patient-derived xenografts were treated with placebo, tamoxifen, CDB4124 or tamoxifen plus CDB4124. Average tumor volume at the start of therapies was 125 mm^3^ and the total tumor volume is shown (*n* > 9). Mean and S.E.M are plotted. (^*^ <0.05 and ns not significant). **G.** In ER/PR crosstalk, distinguishing overlapping effects observed due to concomitant interaction of another receptor with its ligand (agonist or antagonist) from unique isoform and ligand-specific effects would guide the development of biomarkers for patient selection and translation of PR-targeted therapies to the clinic.

The use of functionally diverse PR agonists and antagonists to study PR biology has contributed to the difficulties of side-by-side comparisons in existing studies to understand PR biology. It is difficult to determine whether the observed differences in these studies are due to the differences in PR biology or are due to the functional diversity of various ligands used in these studies. Therefore, a head-to-head comparison of functionally diverse PR agonists, selective PR modulators (SPRMs) and PR antagonists is needed to better understand PR biology and for clinical translation of PR-targeted therapies. To this end, we performed genomic analysis in T47D xenografts treated with various SERMs (tamoxifen, bazedoxifene, raloxifene), selective ER degrader fluvestrant, PR agonists (progesterone, medroxy progesterone acetate (MPA) or synthetic progestin R5020), PR antagonists CDB4124 and CDB4453, and SPRM EC313 (Figure 3A). CDB4124 and CDB4453 are highly-selective PR ligands that antagonize R5020-induced transcriptional activity and proliferation of T47D cells [45–48]. In contrast, EC313 is a SPRM engineered on a PR agonist molecular backbone [47, 48]. In agreement to EC313's mixed agonist and antagonist activity, EC313 is half as effective as the pure PR antagonist EC317 at inhibiting R5020-induced viability of T47D cells (Figure 3B). Unsupervised analysis of transcriptional responses in treated T47D xenografts clustered gene expression into PR agonist or antagonist-specific groups (Figure 3C - 3D and Supplementary Table 4). It is remarkable that despite the variety of PR ligands used, gene expression clustered based on whether PR is activated or inhibited by an agonist or antagonist. The protective/neutral effects of natural progesterone [2, 49–53] compared to synthetic progestins and MPA [29–32] in modulating breast cancer risk is controversial. While some differences in gene expression induced by the three PR agonists could potentially be important, it is noteworthy that a relatively high correlation was observed between gene expression patterns induced in response to these three PR agonists, indicating functional similarities among PR agonists when compared to PR antagonists. Because expression profiles did not cluster by the presence or absence of tamoxifen (Figure 3C), despite ER expression (Figure 3E and 3F), we hypothesized that a higher positive correlation would be observed in the transcriptomes observed in response to joint therapies with a PR-targeting drug and SERMs (tamoxifen, raloxifene or bazedoxifene) or selective estrogen receptor down regulators (SERDs) like fulvestrant. In support of our hypothesis, unsupervised analysis of transcriptional responses in T47D xenografts treated with EC313 plus various SERMs indicated that the transcriptomes cluster based on the presence or the absence of exogenous PR-targeting drug (Figure 3G). The strength of effects of PR on ER activity is likely not due to low ER levels, because the T47D cells used to seed the xenografts expressed easily detectable ER and PR (Figure 3E) and tamoxifen significantly inhibited the growth of these xenografts (Figure 5E). Notably, while different treatment regimens had variable effects on ER and PR expression levels (Figure 3F), these effects were not consistent within the agonist and antagonist groups. These results indicate that although the modulation in receptor levels could be one of the modes of actions of these drugs, the variations in receptor levels alone cannot explain the observed segregation of transcriptomes (Figure 3C and 3D) in PR ligand-type groups. These results are consistent with our prior observations that gene expression patterns upon joint activation of ER and PR correlated significantly with those observed after activation of PR alone, but not ER alone [1], indicating that in these model systems, PR governs estrogen biology.

### PR isoforms differentially reprogram ER binding

**Figure 4:**
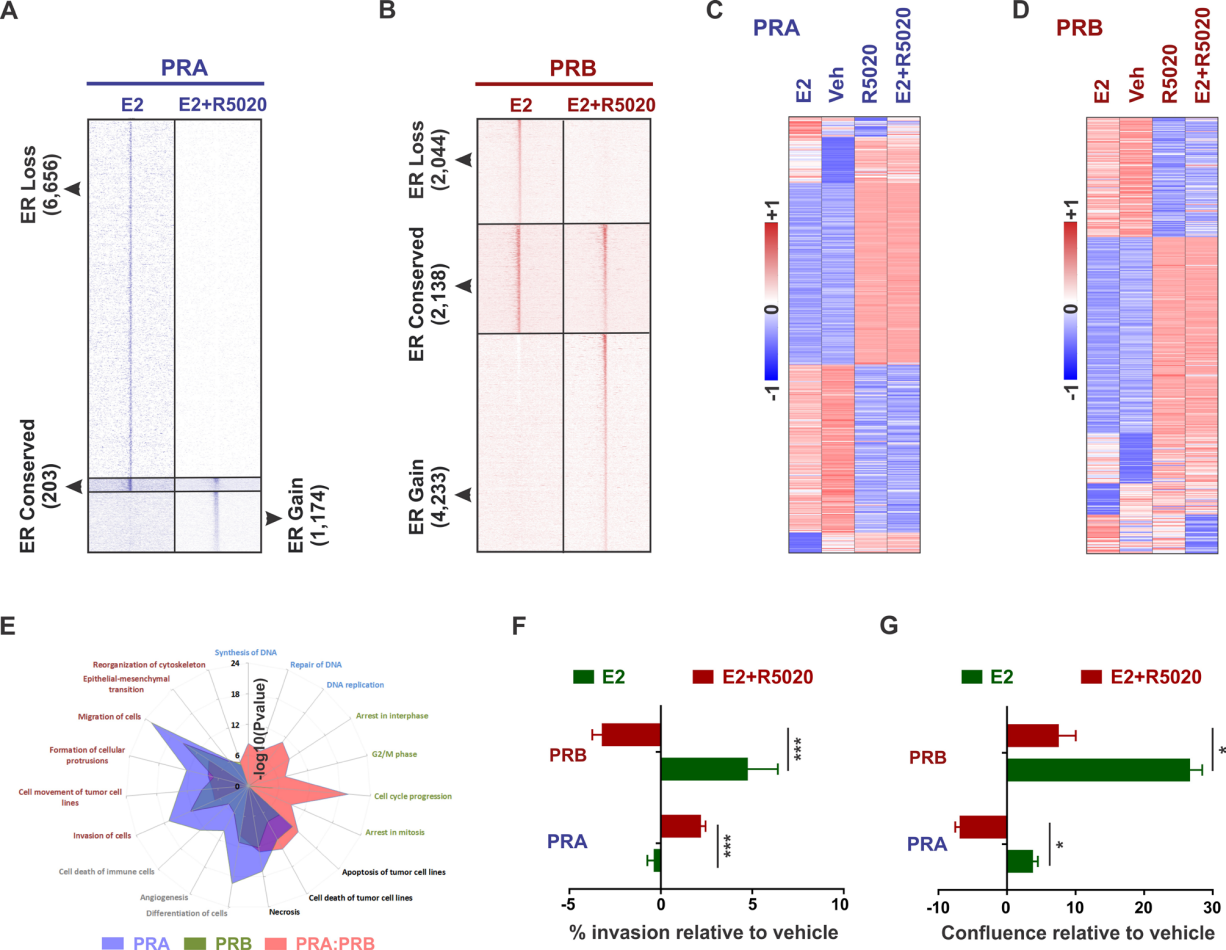
PR isoforms appropriate similar motifs at distinct locations to differentially reprogram estrogen signaling **A.** - **B.** Heatmaps display intensity of sequencing obtained on anti-ER ChIP before (10 nM estrogen (E2) alone) and after (10 nM estrogen plus 10 nM R5020) remodeling by PRA **A.** or PRB **B.**. **C.** - **D.** Expression of estrogen and progestin-regulated genes in T47D cells expressing either PRA **C.** or PRB **D.**. Heatmaps are row-normalized and include the union of estrogen and progestin-regulated genes. **E.** Cellular pathways enriched in the genes that are differentially expressed in response to 10 nM R5020 treatment of T47D cells expressing PRA/PRB mixtures [1] or PRA and PRB individually. **F.** - **G.** Changes in **F.** matrigel invasion and **G.** cell confluence of ER+/PRA+ and ER+/PRB+ T47D cells in response to treatments with 10 nM estrogen or both 10 nM estrogen plus 10 nM R5020. ^*^ denotes *P*-value < 10^-1^, ^***^ *P*-value < 10^-3^. Three biological replicates were performed and each of the experimental conditions had at least 12 technical replicates.

Based on published reports of PR isoform-specific ER/PR crosstalk observed using artificial vector constructs [33–38] and our findings of different biologies for the two PR isoforms in breast cancer cells (Figure 1), we hypothesized that these isoforms differentially reprogram estrogen signaling. In support of our hypothesis, while a PRA/PRB mixture (T47D cell model expressing both the PR isoforms) has been reported to expand ER chromatin binding [1, 2], PRA inhibited ER binding by 70% (Figure 4A), and PRB primarily redistributed ER genomic recruitment (Figure 4B). The negative effects of PRA on ER binding agree with the observed inhibitory effects of PRA on gene expression (Figure 2A and 2B). Importantly, the levels of transcripts observed on joint activation of ER and PR isoform, correlated with the respective PR isoform, but not with ER Figure 4C and 4D). This PR isoform-specific modulation of ER genomic activity was further reflected in the Ingenuity analyses of the isoform-specific transcriptomes (Figure 4E) and the observed phenotypes. In line with the individual phenotypes of the two isoforms (Figure 2C and 2D), PRB decreased estrogen-induced invasion (Figure 4F) and increased estrogen-induced proliferation (Figure 4G), while PRA had the opposite effect on these phenotypes (Figure 4F and 4G). Of note, in similar experiments with wildtype T47D cells (expressing comparable levels of PRA and PRB), 10 nM R5020 in isolation led to a modest increase in cell number and an insignificant change in cell invasion [1]. The magnitude of these phenotypes was much higher under estrogenic conditions, in which 10 nM R5020 abrogated estrogen-induced cell proliferation as well as invasion [1, 2]. Collectively, these results indicate that the relative ratio of PR isoforms in breast cancer cells affects cellular responses to progestogens.

### Combination therapies with tamoxifen and SPRM EC313 or PR antagonist CDB4453 lead to tumor regression

The predominance of PR isoform A has been associated with higher tumor malignancy and patients with PRA-rich tumors have worse prognosis compared to tumors with comparable levels of the two PR isoforms [9, 10, 54]. In agreement with these clinical observations, the observed PRA or PRB-induced gene signatures were associated with significantly poorer (*P* value = 4.3×10^-5^) (Figure 5A) or better (*P* value = 0.05) (Figure 5B) patient survival respectively. The PR isoform ratio is a prognostic marker for responsiveness in breast cancer to PR antagonists. [17, 44, 55] In addition, it has been reported that phospho-PRB induced gene signatures identify PR-driven tumors that might benefit from PR antagonist therapies [56, 57]. However, gaps in our understanding of the intersection of isoform-specific networks with PR agonist and antagonist-induced regulatory networks inhibit optimal patient selection for PR-targeting treatments. To understand the clinical relevance of PR agonist and antagonist therapies, we correlated the expression of agonist and antagonist-induced gene expression (Figure 3C) with survival outcomes in two independent patient cohorts. Interestingly, in both patient cohorts, PR antagonist-associated (Figure 5C and Supplementary Figure 3A), but not PR agonist-associated gene expression (Figure 5D and Supplementary Figure 3B), predicted better survival outcomes. These clinical observations combined with reports that PR antagonists inhibit growth of breast tumors expressing higher levels of PRA [17, 44, 55], a PR isoform associated with poor survival outcomes (Figure 5A), motivated us to study the anti-tumor activity of PR antagonists, alone and in combination with tamoxifen. Joint treatment with tamoxifen and the PR antagonist CDB4453 or SPRM EC313 resulted in the regression of T47D xenografts, while individual therapies inhibited tumor growth with no net regression (Figure 5E). These observations are consistent with our recent report of tumor regression in response to treatment of T47D xenografts with tamoxifen plus the PR antagonist CDB4124 [1]. The observation that PR antagonist-associated, but not PR agonist-associated gene expression, predicted better survival outcomes ((Figure 5C - 5D and Supplementary Figure 3A - 3B)) are reflected in our results showing higher anti-tumor activity upon co-treatment with PR antagonists *versus* the cytostatic effect of the PR agonist progesterone [2]. Stronger repression of ER transcriptional activity by antagonist-occupied PR when compared to agonist-occupied PR [33–35] further corroborates our observations of higher anti-tumor activity for PR antagonists *versus* PR agonists. In agreement with our prior report that PR is necessary and sufficient for progestin-mediated reprogramming of estrogen signaling [1], we observed that the CDB4124 phenotype was absent in an ER+/PR- patient-derived xenograft (PDX) (Figure 5F). These results indicate that PR is required for the actions of CDB4124 on tumor growth.

Others have noted that PR isoforms differentially affect the growth of estrogen-dependent breast tumor xenografts [16]. The current study, in conjunction with the published literature [16, 17, 42–44, 55, 58], suggests a need to better understand the effects of PR agonists and antagonists on xenografts expressing disproportionate levels of the two PR isoforms. Among the PR antagonists, the regression of wildtype T47D xenografts observed with CDB4453, a mono-demethylated metabolite of CDB4124, or SPRM EC313 was lower in magnitude compared to the tumor regression observed with CDB4124 [1], which indicates that different SPRMs have variable anti-tumor activity. Importantly, our observations of differential activities of functionally diverse PR agonists and antagonists suggest a need for careful selection of PR-targeting therapies for breast cancer management.

In conclusion, the collective results shown here that PR isoforms, agonists and antagonists distinctly reprogram estrogen signaling, warrants additional research into appropriate combination therapies to treat ER+/PR+ breast cancers (Figure 5G).

## DISCUSSION

There is significant interest in the possibility of exploiting ER/PR crosstalk to treat and/or prevent ER+/PR+ breast cancers. While ER-targeted therapies are widely used for breast cancer treatment and prevention, the clinical efficacy of PR-targeted therapies either alone or in combination with ER-targeted therapies is not well understood nor has it been adopted as a therapeutic choice. The major roadblocks to clinical translation of PR-targeted therapies have been the paradoxical anti or pro-tumorigenic effects of functionally diverse PR ligands (agonists and antagonists) and an incomplete understanding of PR isoform and ligand-selective ER/PR crosstalk. One of the other difficulties in interpreting the PR literature is that a variety of natural and synthetic progestins, with presumably variable actions, have been used to study PR biology [1, 2, 26, 29–32, 49–52]. Use of functionally diverse PR ligands makes it difficult to compare the results of different studies. For this reason, a head-to-head comparison of regulatory networks of diverse PR agonists (progesterone, MPA and R5020) and antagonists (CDB4124, CDB4453 and SPRM EC313) is essential.

In T47D cells expressing either of the two PR isoforms (PRA and PRB) on a PR-deficient background [13, 16], we demonstrate that each isoform appropriates similar binding motifs (Figure 1B) at distinct but overlapping genomic locations to differentially reprogram estrogen biology to be either pro or anti-tumorigenic. PRA and PRB mostly have isoform-specific cistromes (Figure 1A), interactomes (Figure 1C), transcriptomes (Figure 1D) and functional outcomes (Figure 2C and 2D). Our observations are generalizable beyond the T47D model system used in this study because PR isoform dependence of transcriptomes has been observed in unrelated model systems [40, 41] and to some extent in primary PR+ breast cancers expressing disproportionate levels of the two PR isoforms (Figure 2E and 2F). Of the two isoforms, PRA is a more potent repressor of gene expression (Figure 2A and 2B). While mixtures of PRA/PRB are reported to expand ER binding [1, 2], in agreement with the stronger inhibitory effects of PRA on gene expression, PRA primarily inhibits ER genomic recruitment (Figure 4A) while PRB redistributes ER chromatin binding (Figure 4B). The observed inhibitory effects of PRA confirm the breast cancer relevance of similar observations made using artificial vector constructs transiently transfected in rat uterine cells and 3T3 mouse fibroblasts [33–35], monkey kidney CV-1 fibroblasts and MCF10A cells [36–38]. These results also agree with published literature on the differential biology of the two PR isoforms studied in different breast cancer cell models [6, 9, 14, 40–43]. The two PR isoforms reprogram estrogen signaling to be anti- or pro-tumorigenic such that the transcriptomes (Figure 4C and 4D) and functional outcomes (Figure 2C - 2D and 4F - 4G) on joint activation of ER and PR correlate with those observed upon the activation of each PR isoform alone but not ER alone. The clinical relevance of our PR-isoform specific findings was corroborated by the observations of differential gene expression patterns in the groups of patient tumors expressing higher levels of either PRA or PRB (Figure 2E and 2F). Not surprisingly, the clustering signal observed in patient tumors was relatively weaker indicating that unlike in-vitro models, primary tumors are very divergent and other drivers may confound clustering over PR isoforms ratios. Importantly, in agreement to the published literature [9, 10, 54], the observed PRA or PRB-rich gene signatures were associated with significantly poorer or better survival outcomes respectively (Figure 5A and 5B). Collectively these results underscore the importance of understanding PR isoform-specific biology for the successful targeting of PR in breast cancer management.

To perform head-to-head comparisons of several PR agonists (progesterone, MPA and R5020) and antagonists (CDB4124, CDB4453 and SPRM EC313), we performed genomic studies in T47D xenografts treated with various combinations of PR-targeting therapies alone or in combination with SERMs (tamoxifen, bazedoxifene, raloxifene) and fulvestrant, a potent ER degrader (Figure 3A). Activation or inhibition of PR segregates transcriptomes into separate PR agonist and antagonist-mediated groups (Figure 3C and 3D). The protective/neutral effects of progesterone [2, 49–52] *versus* synthetic progestin R5020 and MPA [29–32] in modulating breast cancer risk are controversial. Although there are some differences between various PR agonists which could be possibly important, the three PR agonists induced relatively similar gene expression patterns (when compared to PR antagonists) in xenografts, suggesting functional similarities among PR agonists when compared to PR antagonists.

The transcriptomes observed in response to co-treatment with SPRM EC313 and either of the three SERMs (tamoxifen, bazedoxifene, raloxifene) or fulvestrant, clustered separately from the ones observed for individual tamoxifen therapy (Figure 3G). These results indicate that in these model systems, PR governs ER-regulated gene expression when both receptors are present and activated. The magnitude of PR influence on ER does not appear to be related to ER expression levels because the T47D cells used for xenograft studies expressed significant levels of ER (Figure 3E) and tamoxifen was an effective inhibitor of tumor growth (Figure 5E). The observations reported here that the relative ratios of PR to ER might not be a limiting factor are in agreement with our prior work in which overexpression of ER or moderate knockdown of PR in T47D cells did not significantly alter the effects of PR on estrogen-regulated gene expression [1]. Although modulation of receptor levels might be one of the modes of actions of the therapies investigated in this study (Figure 3F), it is important to note that factors independent of the variations in receptor levels could also contribute to the observed differences because treatment regimens did not consistently alter the ER/PR levels between the PR agonist and antagonist groups (Figure 3F).

Higher PRA to PRB ratio in breast tumors has been associated with significantly poorer patient survival (Figure 5A and 5B) [9, 14, 54]. PR isoform ratio is a prognostic and predictive factor for responsiveness of breast tumors to PR antagonists and PR antagonists inhibit growth of breast tumors expressing higher levels of PRA [17, 44, 55]. Others have reported that loss of PRB in PRA-rich tumors may signify lower levels of phospho-PRB and have associated phospho-PRB activity with tumor aggressiveness. Post-translational modifications of PR alters PR-regulated gene expression and phospho-PR induced gene expression is reported to identify PR-driven tumors that might benefit from antiprogestin treatments [56, 57]. Therefore, it is not surprising that the gene signatures induced by PR antagonists but not PR agonists were associated with better survival outcomes in two independent patient cohorts (Figure 5C-5D and Supplementary Figure 3A-3B). This differential of better patient survival associated with PR antagonist *versus* agonist treatment is consistent with the observed higher anti-tumor activity of tamoxifen combined with PR antagonists CDB4124 [1], CDB4453 and SPRM EC313 (Figure 5E) *versus* the cytostatic effect observed with the PR agonist progesterone [2]. The higher anti-tumor activity of PR antagonists *versus* PR agonists is further corroborated by the stronger repression of ER transcriptional activity by antagonist-occupied PR *versus* agonist-occupied PR [33–35].

PR agonists and antagonists have been used for treatment of advanced breast cancer with limited success [17, 20–24]. Multiple clinical studies suggest a therapeutic value of high-dose progestins in breast cancer treatment [20–24], although a clear consensus of the therapeutic benefit from dose-escalation studies is lacking. Additionally, it remains to be determined to what extent the therapeutic effect of high-dose progestins is due to an indirect effect *via* the hypothalamic/pituitary/ovary axis. For example, the dual nature of high *versus* low-dose estrogens is well documented and in 1960s and 1970s, high dose diethylstilbestrol was the mainstay in the treatment of ER+ breast cancers [59]. Another concern regarding PR agonist therapies is that although progesterone in combination with estrogen inhibits estrogen-induced proliferation [1, 2], progesterone alone is a mitogen in breast cancer [56], and 10 nM progesterone leads to robust proliferation in ER+/PR+ human tumor explants [57]. Furthermore, under steroid-deprived conditions, there is a high correlation between ER and PR-regulated genes in response to cognate agonists, suggesting that in isolation, both receptors regulate gene expression in similar directions [1, 39]. Thus it is important is to study the long-term effects of high-dose PR agonist therapies because the short term benefit of PR agonist inhibition of estrogen-induced tumor growth might occur at the expense of stem cell expansion to produce receptor-negative and drug-resistant populations, resulting in drug resistance [25, 27, 28, 58, 60].

While PR antagonists may prove to be more effective therapeutically, one of the concerns is that most of the older clinically-tested PR antagonists, such as onapristone and lonaprisan, have been associated with liver toxicity. Thus there is a need to develop potent PR antagonists with minimal toxic effects [17, 61]. Recently, several promising PR antagonists and SPRMs such as CDB4124, CDB4453 [45, 46], EC303, EC317 and EC313 [47, 48] have been developed and it would be worthwhile to study the therapeutic and toxicity profiles of these drugs in humans. Interestingly, unlike the older clinically-tested PR antagonists, oral administration of CDB4124 at 12.5 mg/day does not result in adverse liver toxicity in humans [62]. The higher anti-tumor activity of CDB4124 [1] coupled with a lower liver toxicity profile [62] suggests that safer PR antagonists could potentially be one of the ways forward for breast cancer treatment. CDB4124 (Telapristone acetate) is actively being tested in several clinical trials to treat uterine fibroids as well as in a breast cancer prevention study of high risk women undergoing mastectomies [63, 64].

Therapy selection depends on an in-depth evaluation of both the advantages and disadvantages of the choices at hand. Although PR agonists as well as antagonists have been reported to inhibit estrogen-induced proliferation, it is important to evaluate the costs and benefits associated with the different biologies of the two ligand choices for rational decision making. Importantly, there is a need to differentiate common effects observed due to concomitant interaction of another receptor with its ligand (agonists or antagonists) from unique isoform and ligand-specific effects (Figure 5G). For instance, it remains to be determined how and to what extent the concomitant interaction of another receptor with its agonists or antagonists redistributes ER-associated interactomes. Additionally, a recent study suggests that PR could influence tumor growth independently from direct modulation of ER transcriptional activities [4] thereby emphasizing the value of carefully selecting ligand choices for PR targeting.

In summary, the head-to-head comparisons described here of PR isoforms, PR agonists and antagonists and their intersection with estrogen signaling strongly suggest a need for follow up studies to identify and develop appropriate biomarkers to select breast cancer patients that will benefit from ER/PR-targeted co-therapies.

## MATERIALS AND METHODS

### Xenograft experiments

All mouse studies were carried out under an approved Institutional Animal Care & Use Committee (IACUC) protocol number 70899. Nude mice (J:nu) were obtained from Jackson Labs at an age of 4-6 weeks old. All mice were ovariectomized females with an average weight of 20 grams. Nude mice were anesthetized with isoflourane and an incision was made on the back of the neck. A silastic implant containing 17-β-estradiol was inserted under the skin and several sutures were applied. The 5 mg 17-β-estradiol silastic implants were made as follows: a 1.4 cm portion of silastic tubing (Dow Corning 0.078 in x 0.125 in OD Catalog no. 508-009) was filled with 5 mg 17-β-estradiol (Sigma E2758-1G) and 10mg cellulose (Sigmacell Cellulose Type 20, 20um S3504-500G) and sealed with aquarium glue. Circulating estradiol determinations were made by the University of Chicago clinical laboratory by obtaining blood from mice with implants by cardiac puncture. The estradiol levels of 183 pg/ml, 190 pg/ml, 170 pg/ml, 177 pg/ml and 199 pg/ml were observed in the blood of five ovariectomized mice in response to four weeks of treatment with estradiol silastic implants. None of these mice had bladder issues. These observations agree with previous reports in which 2 mg silastic E2 impants were used in intact and ovariectomized mice for 6-8 weeks [16].

Sufficient numbers of T47D cells were cultured *in-vitro* and at the day of cell injections, the cells were harvested and suspended in PBS. 10 million T47D cells were injected in mammary fat pad along with biodegradable matrigel. For the EC313 experiments, four tumors per mice were grown (left and right hand sides of top and bottom mammary fat pads). Similarly, for the CDB4453 experiments, two tumors per mice were grown (left and right hand sides of the bottom mammary fat pad). From a month of initial cell injections, after the tumors reached 120 mm^3^, the mice were implanted with 25 mg and 90 days release pellets for CDB4453, placebo, tamoxifen, or CDB4453 in combination with tamoxifen (Innovative Research of America). EC313 (alone and in combination with tamoxifen) was administered as 10 mg/kg/day intra-peritoneal injections. Five injections per week were administered. Xenograft tumor size was measured weekly and percentage change in tumor volume since the start of therapy is reported. At the end of the experiment, tumors were excised, weighed and fixed or stored in liquid nitrogen for subsequent analysis. Mice were sacrificed after 35 and 42 days for preclinical studies with EC313 and CDB4453 respectively.

For the studies involving PR agonists (progesterone, MPA and R5020), selective PR modulator EC313, SERMs (tamoxifen, raloxifene or bazedoxifene) and ER degrader fulvestrant, mice were injected intra-peritoneal for three weeks with 10 mg/kg/day of the respective drugs. Five injections per week were administered. At the end of the duration of the experiment, mice were sacrificed; tumors were excised, weighed, fixed and snap-frozen for later analyses. RNA-seq was performed on these harvested xenografts.

PR-targeting therapies CDB4124 and CDB4453 were obtained from Repros therapeutics Inc., Texas, USA. SPRM EC313 and PR antagonist EC317 were obtained from Evestra Inc, Texas, USA. Drugs were obtained from both companies under signed material transfer agreements.

### RNA-seq

Frozen xenograft tumors were finely grinded using cold hammer and a pestle. The pestle was placed over dry ice to maintain it at low temperatures. The ground tumor was suspended in ice-cold PBS and dounce homogenized and subsequently washed twice with ice-cold PBS. The cell pellets obtained from washed xenografts were processed like the pellets obtained from *in-vitro* experiments [1]. There was one mouse (and four tumors) per treatment condition except experiments involving CDB4124 and CDB4453. For experiments involving CDB4124 and CDB4453, there was one mouse (and two tumors) per treatment condition. Except for treatment conditions in which tumor regression was observed, one out of the four tumors were used for RNA-seq experiments. In mice in which tumor regression was observed, multiple tumors had to be combined to ensure enough starting material for RNA-seq.

### Statistical and general methods for animal studies

Power analyses were performed using the power.t.test function of R. The effect sizes and the standard deviations were obtained from our previous preclinical studies using CDB4124 [1]. The sample size calculations were made to ensure 95% power in the experiment with a p value significance of 0.05.

For any given time point, measurements from all the alive animals at that timepoint were included in the results. Measurements from none of the alive animals were excluded for any of the time points. Prior to the start of the experiments, animals were randomized to ensure that the mean starting tumor volumes and the standard deviations in any treatment group are similar. The investigator was not blinded to the group allocation during the experiment.

Package LME4 in R was used to perform linear mixed-effects modeling for calculating the statistical significance of the differences in tumor volumes between the treatment groups. Estimate of variance within each group was performed.

### ER+/PR- patient-derived xenograft experiments

For the Patient-Derived Xenograft model, tumors were initiated *via* serial transfer from a donor mouse bearing breast cancer tumor obtained from Jackson Laboratory (TM00284/BR0853F). After extraction of the tumor from the donor mouse, two mm^3^ tumor pieces were implanted with a trocar in both the left and right inguinal mammary fat pads of intact, non- ovariectomized NSG mice. The tumors were grown according to the protocol from the Jackson Laboratory and they were not supplemented with exogenous estradiol. Tumors were grown until they reached a size of 120 mm^3^, after which the mice were randomized in four groups and treated *via* IP injections with vehicle, tamoxifen (10mg/kg), CDB4124 (10 mg/kg) or both drugs at the same concentration, 5 times/week.

### Patient tumors that are high for either PRA or PRB

Data for nine out of 11 tumors used in this study was obtained from Rojas et al, 2017 [44]. Data for two other unpublished tumors (tumors B3 and B5) used in this study was kindly provided by Dr. Claudia Lanari and Dr. Martin Abba (Laboratory of Hormonal Carcinogenesis, Instituto de Biología y Medicina Experimental (IBYME), Consejo Nacional de Investigaciones Científicas y Técnicas (CONICET), Buenos Aires, Argentina). These unpublished tumors were processed similar to the nine other published tumors [44]. For experimental details and bioinformatics methodology, please refer to Rojas et al, 2017 [44]. The transcriptomic data for these tumors can be accessed from Supplementary Table 3.

### Cell culture

Cells were grown in RPMI 1640 supplemented with 10% fetal bovine serum (FBS) and 1% penicillin streptomycin. T47D cells and derived sublines were provided by Dr. Kathryn Horwitz [13]. Briefly, ER+/PR-low T47D cells were derived from parent ER+/PR+ T47D cells through flow cytometry and PR was stably re-expressed in ER+/PR-low T47D cells to create T47D cells expressing either PRA or PRB isoforms. Media used to grow PRA or PRB-expressing T47D cells was supplemented with 200 μg/ml of geniticin (life technologies, #10131-027) for selection. Prior to experiments, cells were cultured for 48 hours in phenol red free RPMI 1640 supplemented with charcoal-stripped FBS and 1% penicillin streptomycin (steroid-deprived media). Estradiol (Sigma #E8875-250MG) and R5020 (PerkinElmer #NLP004005MG) dissolved in ethanol (vehicle) were used at a final concentration of 10 nM for all the experiments except the cell viability assays. The cell viability assay was performed at various concentrations ranging from 1 pM to 10 nM. STR analyses were performed and the cell line matched T47D cell line at all the listed loci.

### Cell migration (scratch wound) assays and invasion assays

#### Cell migration

T47D cells were grown in 96 well ImageLock plates (Essen Bioscience #4379). After the cells reached 90% confluence, they were deprived of steroids for 48 hours. Thereafter, scratch wounds were made using 96 pin WoundMaker (Essen Bioscience #4493) and washed twice with phosphate buffered saline (PBS). Cells were then treated and the confluence of the wound was analyzed over time using an integrated cell migration analysis module (Essen Bioscience #9600-0012). Wound confluence is expressed as the percentage of the wound area occupied by cells and is plotted 48 hours after drug treatments.

#### Cell invasion

Matrigel (BD biosciences #356231) was dissolved 1:40 in steroid-deprived RPMI 1640 and 50 μl was aliquoted to the bottom of each well of a 96 well ImageLock (Essen Bioscience #4379) plate. Thereafter, the plate was incubated at 37°C for 30 minutes to allow the matrigel to solidify, and excess media was removed. Cells were then plated on the top of the matrigel layer and allowed to grow for 48-72 hours until they reached 100% confluence. Subsequently, scratch wounds were made using a 96-pin WoundMaker (Essen Bioscience#4493) and washed with PBS. Matrigel was then dissolved in steroid-deprived RPMI 1640 containing hormone treatment, and another 50 μl layer of matrigel was applied above the cells. After complete solidification, 200 μl of steroid-deprived RPMI 1640 containing hormone treatment was added to the wells. Confluence of the matrigel invasion was analyzed over time using integrated cell migration analysis module (Essen Bioscience #9600-0012). Matrigel invasion is expressed as the percentage of the matrigel-filled wound area that is occupied by cells. Matrigel invasion represents 48 hours post-treatment.

Three biological replicates were performed for cell migration and invasion experiments. Each of the experimental conditions had at least 12 technical replicates. One out of the three biological replicates have been plotted. P values were calculated using two tailed student t test. In the figures ^*^ denotes *P* value < 0.01; ^**^ *P* value < 0.001; ^***^ *P* value < 0.0001; ^****^ *P* value < 0.00001, # not significant

### Cell viability assays

T47D cell line (ATCC, Manassas, VA), was routinely maintained in RPMI medium with L-glutamine and 5% fetal bovine serum (FBS) (Life Technologies, Grand Island, NY). Cells were maintained in 5% dextran coated charcoal stripped serum (DCC-FBS) (Life Technologies, Grand Island, NY) in the absence of phenol red for 24 hours before treatments with the drugs of interest at the reported concentrations and till the end of experiment. The assay was performed at various concentrations ranging from 1 pM to 10 nM. For determining cell viability, T47D cells were plated in 6-well plates at the density of 30,000 cells per well. The cell culture media was changed and replenished at the third day from the start of the experiment. After six days of treatment the cell numbers were counted using TC-20 automated cell counter (Bio-Rad, Hercules, CA). Standard deviation was calculated with quadruplicates of area counted per treatment.

### Confluence and proliferation studies

Cells were plated in a 96 well plate. After reaching 30% confluence, cells were deprived of steroids for 48 hours and then treated as indicated. The cell confluence was measured over time using the Essen Bioscience Incucyte. Confluence is defined as the percentage of area covered by cells. P values were calculated using two tailed student t test. In the figures ^*^ denotes *P* value < 0.01; ^**^ *P* value < 0.001; ^***^ *P* value < 0.0001; ^****^ *P* value < 0.00001

### Protein expression

Cells were grown to 60-70% confluence and lysed with standard RIPA buffer. Frozen xenograft tumors were finely grinded using cold hammer and a pestle. The pestle was placed over dry ice to maintain it at low temperatures. The ground tumor was suspended in ice-cold PBS and dounce homogenized and subsequently washed twice with ice-cold PBS. The cell pellets obtained from washed xenografts were processed like the pellets obtained from *in-vitro* experiments. The resulting total cell lysate was run on SDS-PAGE gel, transferred on to nitrocellulose membrane and immunoblotted using antibodies for the proteins of interest. Antibodies used for immunoblotting are anti-ER (HC20 from Santa Cruz), anti-PR KD68 (in-house developed) [65], anti-actin (A-2228 from Sigma). KD68 picks up both the PR isoforms in immunoblots as well as immunoprecipitation [1]. Protein expression was normalized to the actin loading control.

### Chromatin immunoprecipitation (ChIP) and ChIP-sequencing

Cells were grown in steroid-deprived RPMI for 48 hours to 80% confluence, before being treated for with the hormones of interest. Cells were then fixed with 1% formaldehyde for 10 minutes and the crosslinking was quenched with 0.125 M glycine for 5 minutes. Fixed cells were suspended in ChIP lysis buffer (1 ml 1M Tris pH 8.0; 200 μl 5M NaCl; 1 ml 0.5M EDTA; 1 ml NP-40; 1 g SDS, 0.5 g deoxycholate) and sheared in the Diagenode Biorupter for 20 minutes (30 second cycles). 100 μl of sheared chromatin was removed as input control. A 1:10 dilution of sheared chromatin in ChIP dilution buffer (1.7 ml 1M Tris pH 8.0; 3.3 ml 5M NaCl; 5 ml 10% NP-40; 200 μl 10% SDS; to 100 ml with H_2_O), 4 μg antibody and 30 μl magnetic DynaBeads were incubated in a rotator at 4°C overnight. Chromatin was immunoprecipitated overnight using anti-ER (Santa Cruz Biotechnology HC-20), anti-PR (in-house made KD68) or rabbit IgG (Santa Cruz Biotechnology SC-2027). Next, the immunoprecipitated chromatin was washed with ChIP wash buffer I (2 ml 1M Tris pH 8.0; 3 ml 5M NaCl; 400 μl 0.5M EDTA; 10 ml 10% NP-40; 1 ml 10% SDS; to 100 ml with H_2_O), ChIP wash buffer II (2 ml 1M Tris pH 8.0; 10 ml 5M NaCl; 400 μl 0.5M EDTA; 10 ml 10% NP-40; 1 ml 10% SDS; to 100 ml with H_2_O), ChIP wash buffer III (1 ml 1M Tris pH 8.0; 5 ml of 5M LiCl; 200 μl 0.5M EDTA; 10 ml 10% NP-40; 10 ml 10% deoxycholate; to 100 ml with H_2_O) and TE (pH 8.0). Elution was performed twice from beads by incubating them with 100 μl ChIP-elution buffer (1% SDS, 0.1 M NaHCO_3_) at 65°C for 15 minutes each. The eluted protein-DNA complexes were de-crosslinked overnight at 65°C in 200 μM NaCl. After de-crosslinking, the mixture was treated with proteinase K for 45 minutes followed by incubation with RNase A for 30 minutes. Finally, DNA fragments were purified using Qiagen PCR purification kit and reconstituted in 50 μl nuclear-free water. Real time PCR was performed using SYBR green. For ChIP-seq library preparations, libraries were prepared using KapaBiosystems LTP library preparation kit (#KK8232) according to the manufacturer's protocol. DNA concentrations in inputs samples were measured using nanodrop and 30 ug of the input DNA was used for library prep. For the eluted ChIP sample, half of the eluted ChIP sample (15 ul out of 30 ul) was used for library preparation. The prepared libraries were quality checked using Agilent bioanalyzer and subsequently the concentrations of the prepared libraries were calculated from their bioanalysis profiles. These measured concentrations of the individual libraries were used to calculate the amounts of DNA in individual libraries and subsequently to ensure that different samples are sequenced in almost equal proportions. For the PCR enrichment step of the library preparation protocol, twelve PCR cycles were performed. Three biological replicates were performed for in-vitro directed qPCR reactions. Results from one of the three experiments are presented in Supplementary Figure 1A and 1B. One of the three biological replicates were sequenced for ChIP-seq.

### Mass spectrometry to identify PRA and PRB-associated cofactors

#### Rapid immunoprecipitation mass spectrometry of endogenous proteins (RIME)

Serum-starved T47D cells expressing either PRA or PRB were grown in steroid-deprived media for 48 hours and treated with estradiol plus R5020 for 45 minutes. At this time, cells were lysed, nuclei were isolated, immunoprecipitated with an anti-PR antibody (Santa Cruz sc-7208) or an IgG control (Santa Cruz sc-2027) antibody, and subjected to RIME analysis as previously described [66]. Two biological replicates were performed each for PRA and PRB-specific pulldown. Four biological replicates for control IgG (two for each of the isoforms) pulldown were performed. Anti-PR SC7208 picks up both the isoforms of PR in immunoblots as well as immunoprecipitation [2].

#### Liquid chromatography-electrospray ionization-tandem mass spectrometry (LC-ESI-MS/MS)

Samples were analyzed by mass spectrometry as previously described [67]. (Tryptic digests (1.5 μg) were loaded directly onto 2 cm C18 trap column (packed in-house), washed with 10 μl of solvent A (5% acetonitrile, 0.1% formic acid), and eluted on a 15 cm long, 75 μM reverse phase capillary column (ProteoPep™ II C18, 300 Å, 5 μm size, New Objective, Woburn MA). Peptides were separated at 300 nL/min over a 180-minute linear gradient from 5% to 35% buffer B (95% acetonitrile, 0.1% formic acid) on a Proxeon Easy n-LC II (Thermo Scientific, San Jose, CA). Mass spectra were acquired in the positive ion mode, using electrospray ionization and a linear ion trap mass spectrometer (LTQ Orbitrap Velos^®^, Thermo Scientific, San Jose, CA). The mass spectrometer was operated in data dependent mode, and for each MS1 precursor ion scan, the ten most intense ions were selected from fragmentation by CID (collision induced dissociation). Other parameters for mass spectrometry analysis included: resolution of MS1 was set at 60,000, normalized collision energy 35%, activation time 10ms, isolation width 1.5, and the +1 and +4 and higher charge states were rejected.

#### Peptide and protein identification

MS/MS spectra were searched against the International Protein Index (Kersey et al., 2004) human (v3.87, 91464 entries) primary sequence database using Sorcerer™-SEQUEST^®^ (version v. 3.5,) (Sage-N Research, Milpitas, CA). Search parameters included semi-enzyme digest with trypsin (after Arg or Lys) with up to 2 missed cleavages. SEQUEST^®^ was searched with a parent ion tolerance of 50 ppm and a fragment ion mass tolerance of 1 amu with fixed Cys alkylation, and variable Met oxidation. SEQUEST results were further validated with PeptideProphet [68] and ProteinProphet [69] using an adjusted probability of ≥0.90 for peptides and ≥0.96 for proteins. Proteins considered for analysis were selected based on the schematic shown in Supplementary Figure 2A.

#### Protein quantification and statistical analysis

Proteins detected by LC-MS/MS were quantified by spectral counting, the total number of MS/MS spectra detected for a protein [70]. Differences in relative protein abundance were assessed using the spectral index [71], based on the formula: (Average pulldown in PRA - average pulldown in PRB) / (Average pulldown in PRA + average pulldown in PRB).

### ChIP-seq analyses

The ChIP-seq analyses were performed using ChiLin, a ChIP-seq pipeline developed at Center for Functional Cancer Epigenomics (CFCE), Dana-Farber Cancer Institute (DFCI). Briefly, short reads were aligned to the HG19 human genome using Bowtie2 [72] and subsequently peaks were called using Model-based Analysis of ChIP-Seq (MACS2) peak caller [73]. MACS2 allows for sensitive and robust prediction of binding peaks because it is based on a dynamic poisson distribution that allows for effective capture of local biases in genomic sequence. Subsequently statistically significant peaks are selected by sub setting based on the false discovery rate and the peak height of the reported peaks.

Mapmaker tool developed at CFCE, DFCI was used to apply various statistical and regression techniques to the processed ChIP-seq data. To perform clustering analyses on a group of samples, a union of all the peaks within that group was generated. Number of aligned reads for each of the samples at each of the peaks within the union was extracted from the read alignment BAM files using BEDtools. To normalize by the length of peak and sequence depth, read counts for each peak was normalized to per kilobase of reads per Million mapped reads. These number of aligned reads were used to build a read count matrix for the group of interest. Subsequently quantile normalization was applied to this read count matrix to control for outliers. Various unsupervised and other machine learning techniques were applied to this composite read count matrix of interest. The sample-sample correlation heatmaps represent the correlation observed between any two samples. High correlation (i.e., correlation coefficient 1) between any two samples is displayed in red and low correlation (i.e., correlation coefficient 0) is displayed in blue. The sample-feature heatmaps represent the signal intensity of a feature for any given sample. The heatmaps obtained from unsupervised clustering include the row as well as column dendograms.

CoverageView, GGPlot2, heatmap.2 and Pheatmap packages in R were used to build ChIP-seq heatmaps. The codes for the Mapmaker (https://bitbucket.org/cfce/mapmaker) and Chilin (https://bitbucket.org/shenglinmei/chilin) tools are available on the bitbucket.

### RNA-seq analyses

The RNA-seq analyses were performed using VIPER, a RNA-seq pipeline developed at CFCE, DFCI. Briefly, short reads were aligned to the HG19 human genome using STAR [74]. Subsequently cufflinks packages were used to perform transcript assemblies. Downstream differential gene expression calling was performed using DESeq.

To perform clustering analyses on a group of samples, a union of all the genes and their expression RPKM values within that group was generated to build a read count matrix for the group of interest. Various unsupervised and other machine learning techniques were applied to this composite read count matrix of interest. The sample-sample correlation heatmaps represent the correlation observed between any two samples. High correlation (i.e., correlation coefficient 1) between any two samples is displayed in red and low correlation (i.e., correlation coefficient 0) is displayed in blue. The sample-feature heatmaps represent the signal intensity of a feature for any given sample. The heatmaps obtained from unsupervised clustering include the row as well as column dendograms.

GGPlot2, heatmap.2 and Pheatmap packages in R were used to build various RNA-seq heatmaps. The codes for VIPER is available on the bitbucket (https://bitbucket.org/cfce/viper).

### Patient survival analysis

Patient survival analyses were performed on METABRIC's discovery and validation cohorts. For PR isoforms analyses, a list of top 1000 differential genes were obtained by unsupervised clustering of transcriptomes observed in response to treatments of PR-deficient T47D cells expressing PRA or PRB with various combinations of estrogen and progestin (Figure 1D and Supplementary Table 3). Patient tumors were then segregated (upper and lower quartiles) based on high or low expression of genes specific to each of the isoform clusters. Tumors expressing high or low levels of PRA-specific genes were categorized as “PRA-signature high” or “PRA-signature low” respectively. Similar classification was done based on PRB-specific genes. Subsequently, overall survival curves were plotted for each of the patient groups. Survival package in R was used to perform the analyses. For PR-agonist and PR-antagonist specific gene signatures, similar analyses were done except the list of top 100 differential genes were obtained upon unsupervised clustering of transcriptomes observed in T47D xenografts treated with various combinations of clinically-relevant ER and PR-targeting drugs (Figure 3C and Supplementary Table 4).

## SUPPLEMENTARY MATERIALS FIGURES AND TABLES










