# Supplementary material for: Progesterone receptor isoforms, agonists and antagonists differentially reprogram estrogen signaling
Source: Oncotarget. 2017 Sep 28;9(4):4282–300. doi: 10.18632/oncotarget.21378 (PMC5796974; doi:10.18632/oncotarget.21378)
Supplement: Supplementary file 1 [file oncotarget-09-4282-s001.pdf]

Progesterone receptor isoforms, agonists and antagonists differentially reprogram estrogen signaling

SUPPLEMENTARY MATERIALS

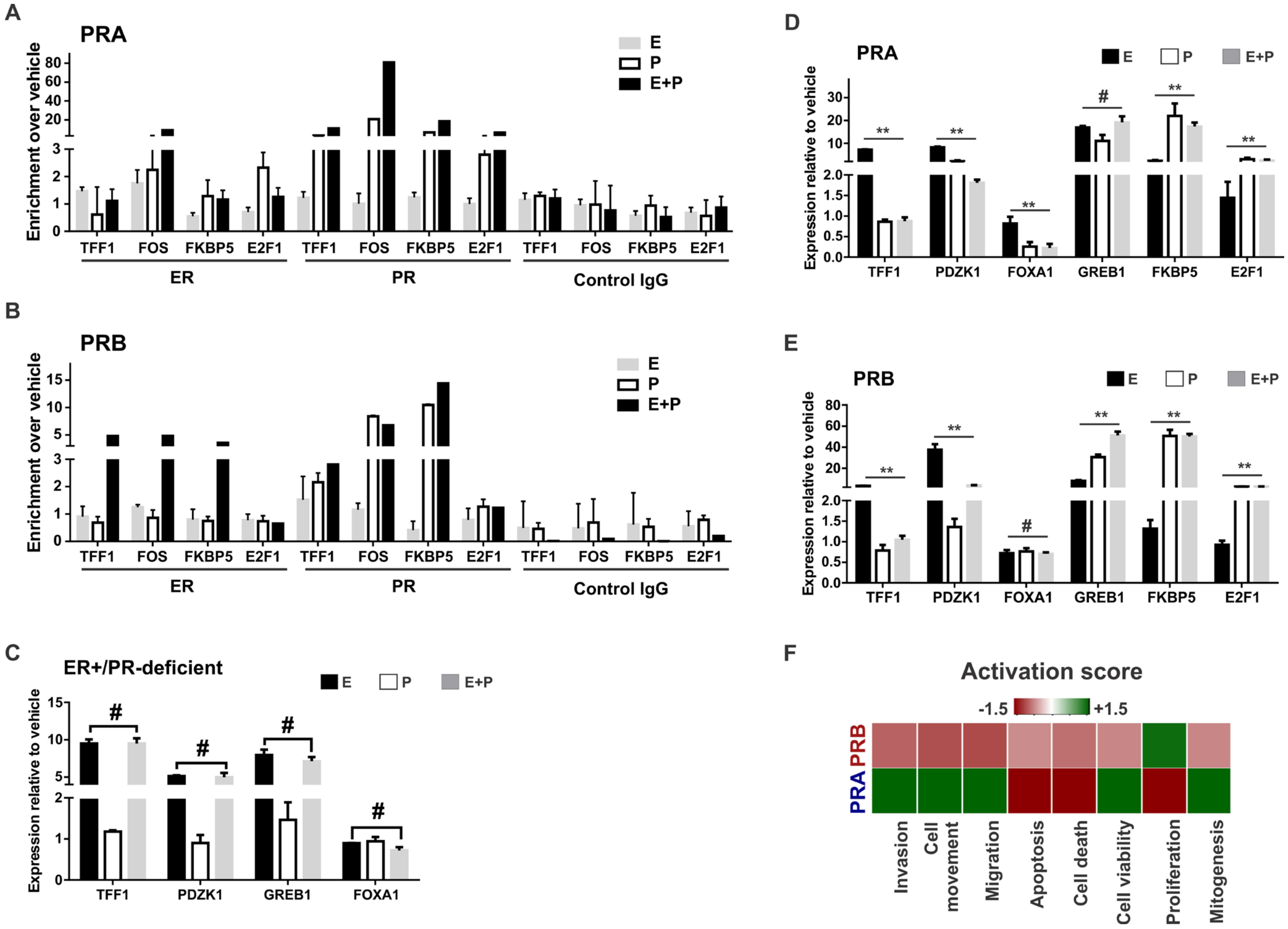

Supplementary Figure 1: PR isoform-specific genomic binding and gene expression. (**A - B**) ChIP-qPCR at select sites in response to 45 minutes of hormonal treatments of T47D cells expressing either PRA (A) or PRB (B). Three biological replicates were performed for in-vitro directed ChIP-qPCR. Results from one of the three experiments are presented. (**C - E**) Estrogen and progestin-regulated gene expression in T47D cells that are either (C) deficient for PR or stably express either (D) PRA or (E) PRB on this PR-deficient background. Three biological replicates were performed for real time PCR reactions. Graphs represent the mean and standard errors of the three biological replicates. (**F**) Strength and direction (Z scores) of activation of functional processes by PRA and PRB-regulated transcriptomes in T47D cells. In the figures * denotes P value < 0.01; ** P value < 0.001; and # not significant.

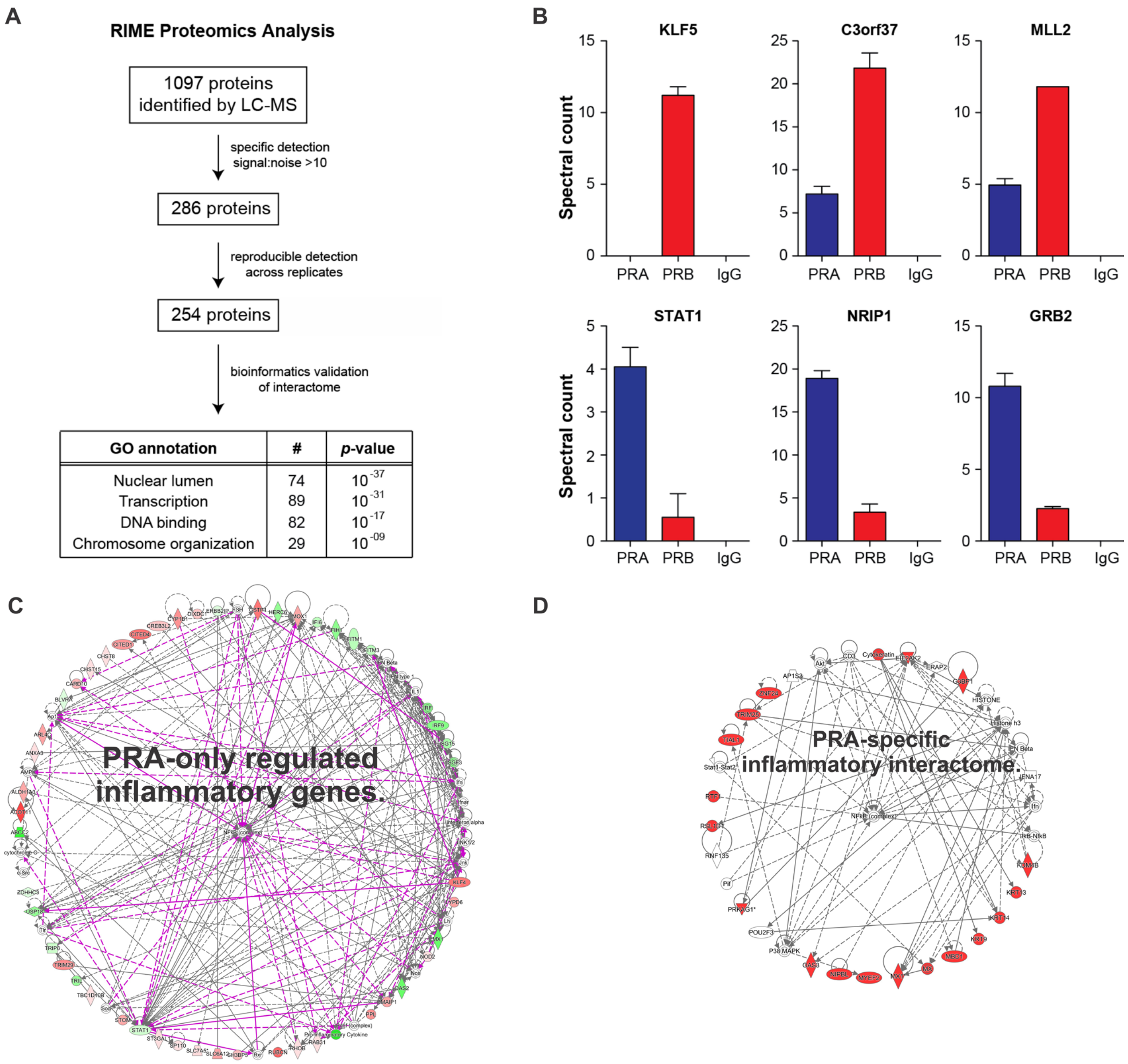

Supplementary Figure 2: PR isoform-specific cofactor recruitment. (**A**) Workflow followed to analyze mass spectrometry data obtained after immunoprecipitation of PRA or PRB. The numbers stated in the workflow represent proteins enriched in PRA and PRB-specific immunoprecipitation. (**B**) Six representative proteins that are differentially enriched in PRA and PRB-specific interactomes. (**C**) Pro-inflammatory pathways were enriched in genes that are differentially regulated by PRA relative to PRB. (**D**) Pro-inflammatory pathways are enriched in coregulators that preferentially interact with PRA but not PRB.

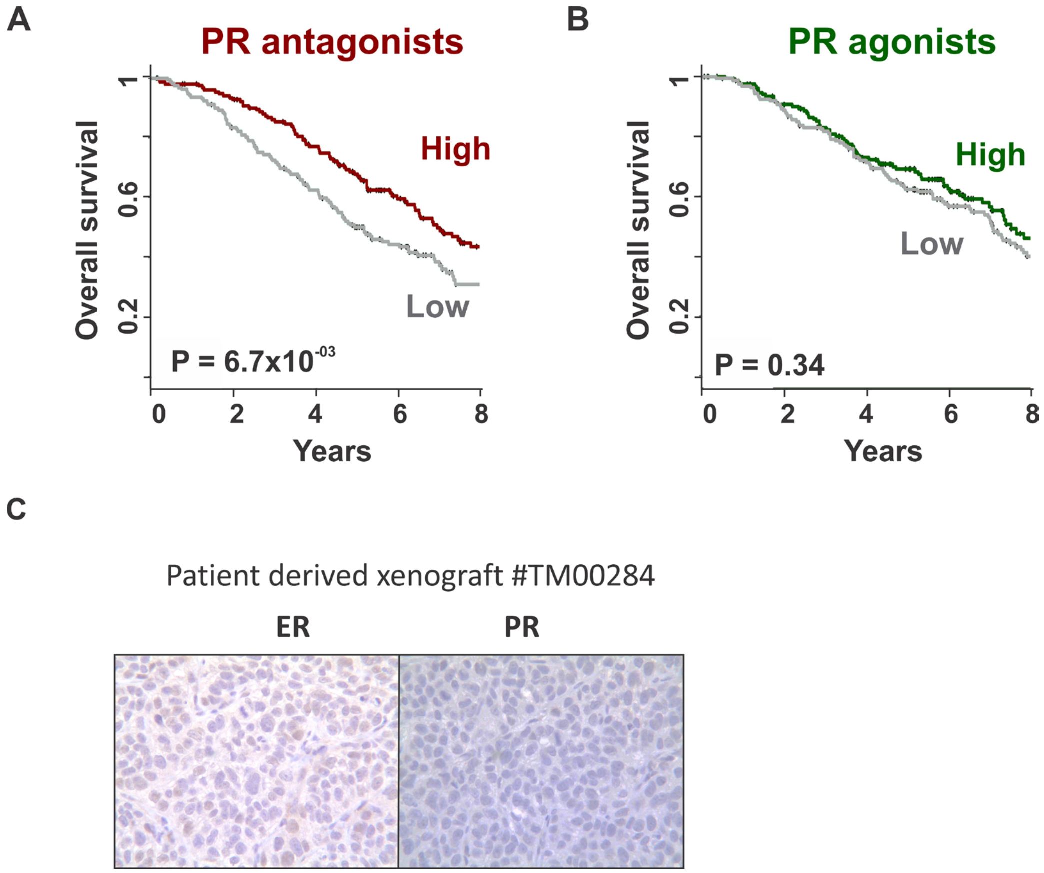

Supplementary Figure 3: Correlation of PR agonists and antagonists-induced gene expression to patient survival outcomes. (**A - B**) Overall survival in METABRIC's validation cohort as classified by high or low expression of (**A**) PR antagonists or (**B**) PR agonists-regulated genes. Agonist and antagonist-regulated genes were obtained from Figure 3C and Table S4. (**C**) Anti-ER and anti-PR immunohistochemistry to determine ER and PR levels in ER+/PR- patient-derived xenograft model.

For Supplementary Tables see in Supplementary Files.

Supplementary Table 1: Differential genomic binding of PR isoforms PRA and PRB. Top 5000 most differential ER and PR binding sites in ER+ T47D cells expressing PRA or PRB. ChIP-seq was performed in steroid-deprived cells treated for 45 minutes with various combinations of 10 nM estradiol, 10 nM R5020 or both the hormones. Tables also include motifs enriched at the binding sites for only PRA, only PRB or overlapping sites for both the receptors. –Log10(P) depicts the significance for the enrichment of a hormone response element in the binding sites of interest.

Supplementary Table 2: Mass spectrometry of cofactors pulled down upon immunoprecipitation of PRA or PRB. Immunoprecipitation for either of the two PR isoforms was followed by mass-spectrometry to identify the isoform-specific recruitment of coregulators. Enrichment of a coregulator for PRA versus PRB is denoted by the metric SI (A:B). Positive and negative values indicate enrichment of a coregulator for PRA and PRB respectively.

Supplementary Table 3: Differential gene expression in T47D cell models and patient tumors expressing relatively higher levels of PRA or PRB. Gene expression in five patient tumors expression higher PRA versus PRB and six tumors with higher expression of PRB versus PRA. Data for nine out of 11 tumors was obtained from Rojas et al (38). Data for two other unpublished tumors (tumors B3 and B5) used in this study was kindly provided by Dr. Claudia Lanari and Dr. Martin Abba.

The files also include data for differential gene expression in ER+ T47D cells expressing PRA or PRB. RNA-seq was performed in steroid-deprived cells treated for 12 hours with various combinations of 10 nM estradiol, 10 nM R5020 or both the hormones.

Supplementary Table 4: Gene expression in xenografts treated with various combinations of ER and PR-targeting drugs. Gene expression in T47D xenografts treated with various combinations of various SERMs (tamoxifen, bazedoxifene, raloxifene), selective ER degrader fluvestrant, PR agonists (progesterone, medroxy progesterone acetate (MPA) or synthetic progestin R5020), PR antagonists CDB4124 and CDB4453 and SPRM EC313.
